# The Impact of Residences and Roads on Wind Erosion in a Temperate Grassland Ecosystem: A Spatially Oriented Perspective

**DOI:** 10.3390/ijerph20010198

**Published:** 2022-12-23

**Authors:** Zhuoli Zhou, Zhuodong Zhang, Wenbo Zhang, Jianyong Luo, Keli Zhang, Zihao Cao, Zhiqiang Wang

**Affiliations:** 1 State Key Laboratory of Earth Surface Processes and Resource Ecology, MOE Engineering Research Center of Desertification and Blown-Sand Control, Faculty of Geographical Science, Beijing Normal University, Beijing 100875, China; 2 College of Surveying and Geo-Informatics, North China University of Water Resources and Electric Power, Zhengzhou 450046, China

**Keywords:** wind erosion, residence, road, temperate grassland, ecosystem management

## Abstract

The existence of residences and roads is an important way in which human activity affects wind erosion in arid and semiarid environments. Studies assessing the impact of these elements on wind erosion have only focused on limited plots, and their threat of erosion to the surrounding environment has been ignored by many studies. This study was based on spatially overlayed analysis of independent wind erosion distribution simulated by the revised wind erosion equation (RWEQ) and remote-sensing-image-derived residence and road distribution data. Wind erosion at different distances from residences and roads was quantified at the landscape scale of a typical temperate grassland ecosystem, explicitly demonstrating the crucial impacts of both elements on wind erosion. The results showed that wind erosion weakened as the distance from residences and roads increased due to the priority pathways of human activities, and the wind erosion around the residence was more severe than around the road. Human activities in the buffer zones 0–200 m from the residences most frequently caused severe wind erosion, with a wind soil loss of 25 t ha^−1^ yr^−1^ and a wind soil loss of approximately 5.25 t ha^−1^ yr^−1^ for 0–60 m from the roads. The characteristics of wind erosion variation in the buffer zones were also affected by residence size and the environments in which the residences were located. The variation in wind erosion was closely related to the road levels. Human activities intensified wind erosion mainly by affecting the soil and vegetation around residences and roads. Ecological management should not be limited to residences and roads but should also protect the surrounding environments. The findings of this study are aimed towards a spatial perspective that can help implement rational and effective environmental management measures for the sustainability of wind-eroded ecosystems.

## 1. Introduction

Wind erosion is an essential process of land degradation and environmental pollution in arid and semiarid areas [1,2]. It severely restricts the extent of land utilization and the sustainable development of society associated with human well-being [3]. Both climate change and human activities can exacerbate wind erosion [4]. Climate change is difficult to address, while human activities are practically flexible through ecosystem management [5]. To effectively control wind erosion, anthropogenic impacts on wind erosion have attracted increasing attention [6].

Human activities change the ecological environment and often result in intensive soil erosion [7]. The existing studies on soil erosion aggravated by human activities mainly include irrational cultivation [8,9] and overgrazing [10] due to their distinct influences on land, such as roughness reduction [11] and crust destruction [12]. In addition to these human activities, wind erosion is also affected by other rural land uses [13], such as residences and roads, but few studies have been conducted on such subjects.

The residence is an essential part of rural land use, and frequent human activity has led to soil erosion in the areas surrounding the residence. Such activity includes the destruction of surface vegetation by human and livestock activities around the residences, changes in soil’s physical and chemical properties due to anthropogenic milling and cutting of topsoil [14], and even destruction of the soil structure by the excavation of the surrounding soil layers [15]. In this disturbed environment, soil erosion readily occurs. For example, in a tropical village in the Lake Victoria Basin, the average soil loss rate of the compounds can reach 107 t ha^−1^ yr^−1^ [16]. On the Loess Plateau of China, rural settlements have severe erosion intensity, with a soil erosion rate of 54.34 t ha^−1^ yr^−1^, which is much higher than that in nonresidential areas [17]. Moreover, the soil erosion modulus caused by cave houses in the Loess Plateau is 4.5 t yr^−1^ per capita, which is 182% more than the soil loss in the primitive environment [18]. Human living activities not only cause water soil erosion, but also induce the formation of blowouts by strong winds in arid and semiarid areas [14]. These studies show that the environment around the residences is vulnerable to soil erosion.

The roads connected to residences are also significant factors affecting wind erosion. Unpaved roads are an important source of dust emissions [19,20]. The surface of the road without vegetation protection is exposed to strong winds [21], and the unpaved road squeezed by wheels is composed of a large amount of loose clay and silt materials [22] and is more susceptible to wind erosion. The road surface is lower than the surrounding grassland and agricultural land, with wind erosion rates of approximately 2.9–3.8 cm yr^−1^ caused by vehicles [23], and the emission potential of PM_10_ increases by 9–160 times under the disturbance of human activities such as traffic [24]. Furthermore, roads also have an impact on the vegetation and soil structures in the surrounding area [25], which shows that the erosion intensity decreases with the distance from the road [26,27,28]. These studies illustrate that the distribution of roads exacerbates regional erosion and dust emissions, which are affected by road characteristics.

Although the area of residences and roads in a region represents a small proportion of the ecological environment, its contribution to soil loss of the whole area is critical [16]. Existing studies have mainly focused on the distribution of residences and roads accelerating water soil erosion, while few studies consider the impact of residences and roads on wind erosion in arid and semiarid environments that are sensitive to human activities. The limited number of studies regarding wind erosion contributed by residences or roads only focused on a few plots [19,20,23], and their impact on the wind erosion of the surrounding areas is rarely determined from a spatial perspective. The spatially oriented approach is essential for a systematic and comprehensive assessment of the scope of human impact on grassland ecology, which allows the focus of ecological management from the point upscale to the landscape. Plot-scale research cannot be extended to the surrounding environment to demonstrate the spatial variation in soil erosion, and regional-scale research often has a coarse spatial resolution, and it is difficult to identify the detailed variation, especially for unpaved rural roads that are prone to erosion. The landscape scale has the advantage of finely investigating the erosion around residences and roads in a continuous and comprehensive environment. Conducting field investigations at the landscape scale combined with remote sensing and GIS spatial analysis can provide a new perspective for quantifying the impact of residences and roads on regional soil wind erosion.

Temperate grassland ecosystems are an important source of dust resulting from wind erosion. The Xilingele grassland is located in the agro-pastoral ecotone and is a part of the wind-eroded areas in northern China [29]. It was found that the increased intensity of human activities caused extensive soil degradation [10]. The study of the influence of rural residences and roads on surrounding wind erosion in this area is of considerable significance to the ecological management in the agro-pastoral ecotone.

To systematically assess the impact of these elements on wind erosion, this study identified all residences and roads in remote sensing images at the landscape scale of the Xilingele grassland and combined them with the wind soil loss determined by the revised wind erosion equation (RWEQ) for systematic spatial analysis. The aims of this study are to (i) quantify the variation in wind erosion along with the distance from residences and roads, (ii) explore the mechanism of the impact of human activity pathways on wind erosion, and (iii) provide a reference for ecological management in arid and semiarid areas.

## 2. Materials and Methods

### 2.1. Study Area

**Figure 1:**
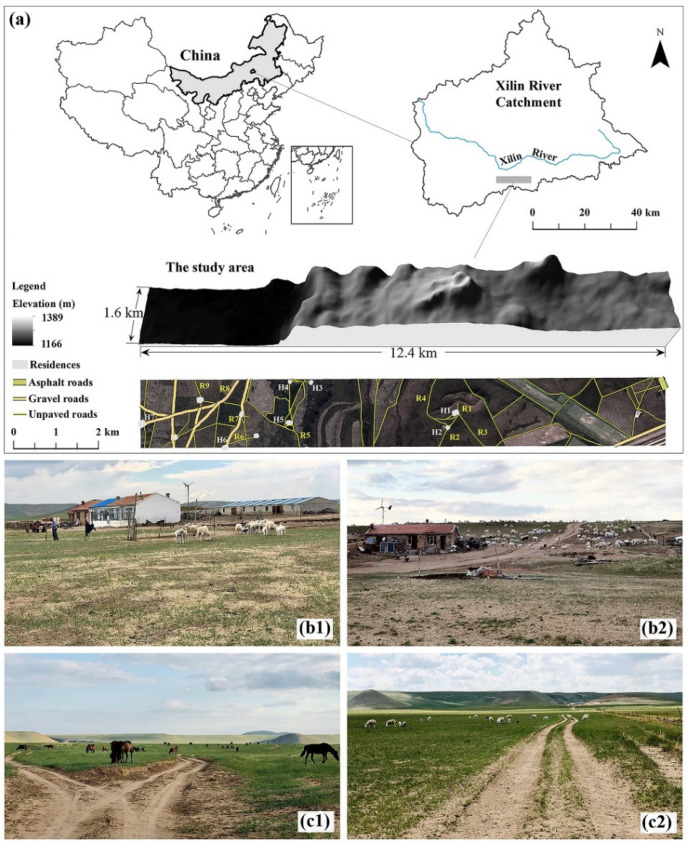
The location, topography, distribution of residences and roads (**a**), and eroded sites around residences (**b**) and unpaved roads (**c**) in the study area.

The study area is located in the Xilingele League, the middle part of the Inner Mongolia Autonomous Region, China (Figure 1a). It has a total area of approximately 20 km^2^, with an east–west length of 12.4 km and a north–south width of 1.6 km. The altitude of the study area varies from 1160 to 1390 m above sea level, and its geomorphological features include flat grasslands in the west and typical hills in the east. The study area belongs to a semiarid continental climate with an average annual temperature of 0.7 °C and annual precipitation of 350 mm. Spring is the period with strong winds, and the prevailing wind directions are west and northwest. The monthly average wind velocity is 3 m s^−1^, and the maximum wind velocity could be greater than 25 m s^−1^, which makes the study area vulnerable to strong winds [30]. The major soil is Kastanozem according to the World Reference Base for Soil Resources [31]. The main vegetation is *Stipa grandis* and *Leymus chinensis*, and the growth period is consistent with the rainy season.

The study area belongs to the agro-pastoral ecotone, which is sensitive to climate change and human activities. The land use is dominated by grasslands with different grazing intensities, and there is also a small part of arable land. Wind soil loss is a critical environmental issue in the study area and is affected by human activities [30]. Field investigations and model simulations found that wind erosion had a strong spatial variation with wind erosion hotspots [32], and wind erosion was related to the distribution of residences and roads. Residences are distributed in the western and central parts of the study area (Figure 1a). The survey found that most residences are accompanied by large sheep pens (Figure 1b). The study area contains three types of roads: the only asphalt-paved provincial road is located in the northeast corner; the trunk roads paved with gravel, accounting for 16.7% of the total road length, are distributed in the western flat area; and the unpaved village roads are distributed throughout the entire area, covering 82.79% of the total length of the road [33]. The first two roads are mainly used for transportation and are fenced, while unpaved roads are used to facilitate grazing.

### 2.2. Simulation of Wind Soil Loss

To systematically evaluate the impact of residences and roads on wind erosion, the spatial variation map of wind soil loss was superimposed with the layers of residences and roads, and the wind soil loss at different distances from residences and roads was extracted. The spatial variation of wind erosion in the study area was simulated by Zhou et al. [32] based on RWEQ, and the calculation formulas are as follows [34]:

(1) $$\mathrm{SL}=\frac{2x}{s^{2}}Q_{\max}e^{-(\frac{x}{s}{)}^{2}}$$

(2) $$Q_{\max}=109.8\left(WF\times EF\times SCF\times K^{\prime }\times COG\right)$$

(3) $$s=150.71{\left(WF\times EF\times SCF\times K^{\prime }\times COG\right)}^{-0.3711}$$

where *SL* is wind soil loss (kg m^−2^ yr^−1^); *x* is the distance to the upwind edge of the field (m); *Qmax* is the maximum soil transport amount (kg m^−1^); and *s* is the field length when the maximum soil transfer reaches 63.2%. *WF*, *EF*, *SCF*, *COG,* and *K′* are five important factors that determine soil wind erosion. *WF* indicates the weather factor, which comprehensively reflects the interaction of meteorological factors. *EF* and *SCF* are the soil erodible fraction and soil crust factors, respectively, which can be calculated by the parameters of the soil’s physical and chemical properties. *COG* represents the combined crop factors, reflecting the protection of crop stubble and vegetation cover on the topsoil by weakening wind. *K’* is the surface roughness factor, which generally refers to changes in the surface caused by tillage that affect wind erosion. The RWEQ can be effectively applied to wind erosion simulations but performs poorly for wind erosion dynamics processes, which can be improved by numerical methods [35,36,37]. Computational fluid dynamics (CFD) wind modelling was integrated into the calculation of the *WF* to enhance the prediction of RWEQ by considering the spatial variation of wind erosion forces. The other wind erosion parameters such as vegetation, soil, and roughness required in the model are based on field measurements and sampling. The parameter collection and simulation process of this study was performed by Zhou et al. [32].

### 2.3. Data Analysis

Based on remote sensing images with a spatial resolution of 2 m downloaded from Google Earth, residences and roads were digitized in ArcGIS 10.2 by visual image interpretation to determine the distribution of residences and roads [33]. Multiple ring buffer analysis tools in ArcGIS 10.2 were applied to generate buffer zones for all residences and roads digitized. The maximum buffer distances of residences and roads were determined to be 3000 m and 600 m, respectively [33]. Areas closer to residences and roads generally experience greater disturbance from human activities. Therefore, the buffer intervals within 1000 m from the residence were 100 m, the intervals between 1000 m and 3000 m were 500 m, and a total of 14 buffer zones surrounded the residences; the buffer intervals within 300 m of the road were set as 30 m, the intervals between 300 m and 600 m were 100 m, and a total of 13 buffer zones were on both sides of the road. The spatial analysis tool in ArcGIS 10.2 was used to extract the wind soil loss at separate distances from residences and roads based on the multiple ring buffer zones. To further explore the mechanism of residences and roads affecting wind erosion, five wind erosion factors (*WF*, *EF*, *SCF*, *COG,* and *K*’) for different buffer distances were also extracted.

The statistical characteristics of the wind soil loss and wind erosion factors of each buffer zone were described, and the significance of the difference between buffer zones was tested using analysis of variance (ANOVA), which was performed in SPSS 26 and plotted in Origin 2018.

The above interval buffers were set according to the size of residences and the level of roads, extracting the wind soil loss in the buffer zones and analyzing the variation characteristics of wind erosion with distance. To explore the impact of residence size on surrounding wind erosion, 18 residences were divided into three areas: more than 4000 m^2^ (large residence), 1000–4000 m^2^ (medium residence), and less than 1000 m^2^ (small residence). The roads were divided into three levels: asphalt roads, gravel roads, and unpaved roads, and the impact of road levels on wind erosion was analyzed.

## 3. Results

### 3.1. Wind Erosion of Residence and Road Buffer Zones

**Figure 2:**
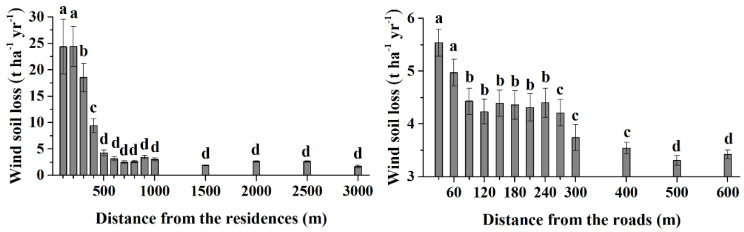
Wind soil loss of Kastanozem at separate buffer distances from residences and roads. Lowercase letters indicate the differences in wind soil loss for different buffer distances (*p* < 0.01).

**Figure 3:**
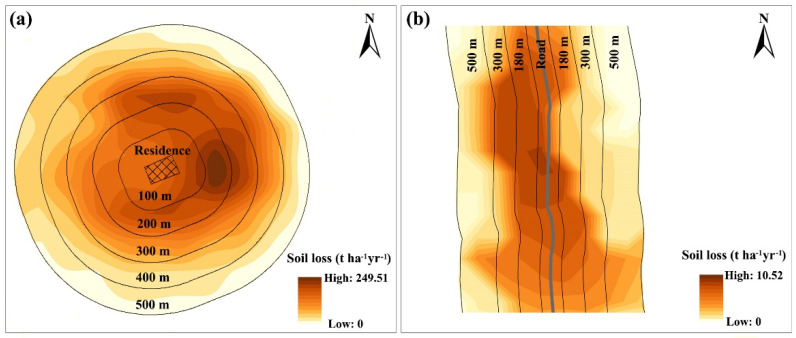
Cases of the spatial distribution of wind soil loss in typical residence (**a**) and road (**b**) buffer zones.

The average wind soil loss at different buffer distances from residences and roads is shown in Figure 2. As the buffer distance increased, the wind soil loss gradually decreased. Wind erosion was the most severe within 200 m around the residences, and the wind soil loss was approximately 25 t ha^−1^ yr^−1^. The erosion intensity decreased significantly to approximately 18 t ha^−1^ yr^−1^ at a distance of 300 m from residences (*p* < 0.01) and to approximately 10 t ha^−1^ yr^−1^ at a distance of 400 m (*p* < 0.01). However, in the range of 500–3000 m from residences, the wind soil loss was less than 5 ha^−1^ yr^−1^. Although the variation trend of wind erosion along residences and roads was similar, the inducing effects of residence on wind erosion were stronger than those of roads. The most severe wind erosion along the roads occurred within 60 m, and the wind erosion in the range of 90–240 m was significantly less than that within 60 m (*p* < 0.01). Furthermore, the wind erosion further weakened with distances from roads greater than 270 m. Figure 3 shows the wind erosion in selected typical residence and road buffer zones. With residence as the center, the wind erosion intensity decreases in a circular ring to the outer circle, and with the road as the center, the wind erosion intensity decreases in a strip shape to both sides.

The percentages of wind erosion intensity in different buffer zones of residences are shown in Table 1. As the distance from residences increased, the proportion of tolerable and slight erosion generally rose, eventually accounting for the majority. The distances of the farthest buffer zone affected by residences with moderate, severe, very severe, and destructive wind erosion intensities were 1000 m, 600 m, 500 m, and 400 m, respectively. The percentages of different wind erosion intensities along the distance from the roads are shown in Table 2. The road buffer zone mainly experienced tolerable and slight erosion, with the proportion of tolerable erosion decreasing slightly with increasing distance, with the opposite trend showing for slight erosion. There was a tendency to remain unchanged in the proportion of moderate erosion. The furthest distances of road buffer zones that suffered severe, very severe, and destructive erosion were 500 m, 400 m, and 300 m, respectively. The above findings indicated that the closer the buffer zone was to residences and roads, the greater the proportion of severe, very severe, and destructive erosion.

**Figure 4:**
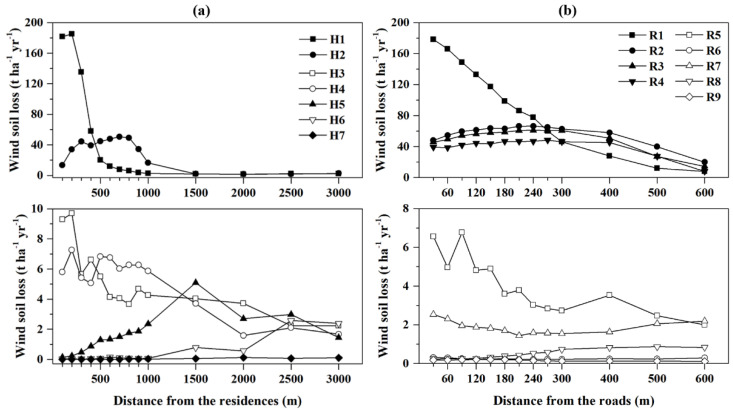
Variation in wind soil loss for individual representative residences (**a**) and roads (**b**) at different buffer distances.

Although wind erosion shows a general increasing trend as it nears residences, there are differences in the characteristics of wind erosion changes around individual residences (Figure 4a). The wind erosion characteristics along the buffer zone of individual residences in different environments (Figure 1a) are distinct. For example, residences located in the central wind erosion hotspots, such as H1 and H2, suffered much more severe wind erosion than other western residences. In the hilly area of the west, the variations in wind erosion around H3 and H4 were consistent with the general trend, reflecting that the wind erosion fluctuated and decreased with increasing distance from the residences. Conspicuously, the wind erosion increased slightly with increasing buffer distance around some residences, such as H5 and H6, which was distinct from the general trend. Wind erosion around other residences in the western plain area was slight, and the variation was weak. For example, there was no wind erosion change for H6 within 1000 m, and wind erosion increased slowly beyond 1000 m; wind erosion for H7 was close to zero in all buffer zones. Therefore, the wind erosion variation in the residence buffer zones in the western area with weak wind erosion was slight, while the variation characteristics of the residence buffer zones in the central hilly area were obvious.

The individual unpaved roads in different environments showed differences in the trend of wind erosion variations along the buffer zone (Figure 4b). For some roads in areas with severe wind erosion, the wind soil loss dropped sharply along with the buffer distance, such as R1. Moreover, wind erosion increased slightly within a range of 300 m along the road, and wind erosion weakened rapidly beyond this range. For example, wind erosion around roads R2, R3, and R4 all had similar variation characteristics. However, there were some cases where the wind soil loss increased along the road buffer zones, such as in R7 and R8. Some roads in areas with weak wind erosion had wind erosion less than 1 t ha^−1^ yr^−1^, and there was almost no wind erosion variation along the road buffer zones, such as R6 and R9. In areas with severe wind erosion, the wind soil loss along the road buffer zone decreased; however, in areas with wind soil loss less than 2 t ha^−1^ yr^−1^, there was almost unchanged wind erosion in the buffer zones.

### 3.2. Wind Erosion Factors of The Residence and Road Buffer Zones

**Figure 5:**
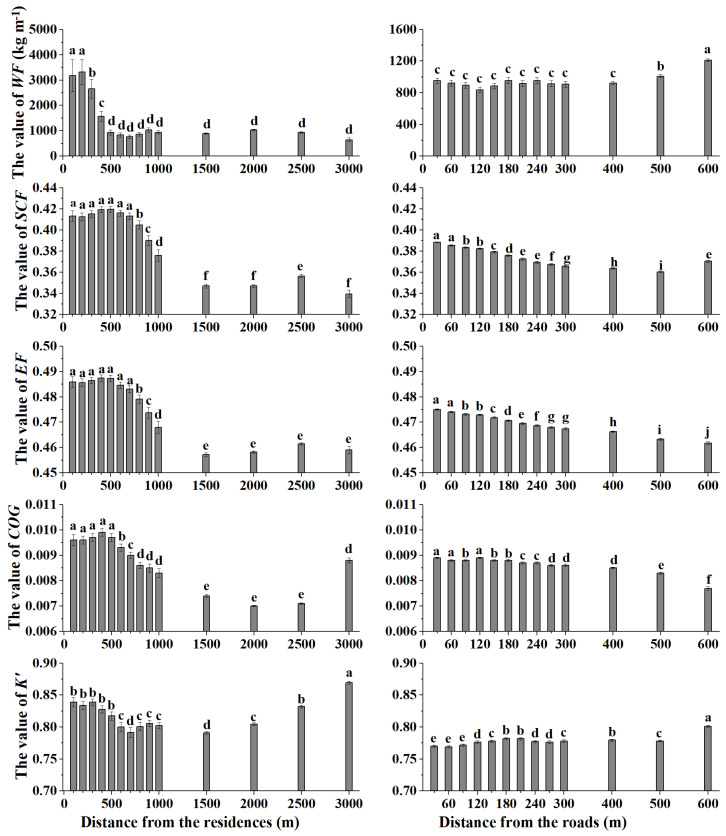
Variation in wind erosion factors (*WF*, *SCF*, *EF*, *COG,* and *K’*) for different buffer distances from residences and roads. Lowercase letters indicate the differences in each wind erosion factor for different distances (*p* < 0.01).

The effect of residences on wind erosion factors is shown in Figure 5. The value of WF decreased gradually from the residences, and the change was slight when the distance was greater than 500 m. The range of disturbance to *SCF* and *EF* from residences was 700 m, and the value of *SCF* and *EF* decreased significantly beyond this distance (*p* < 0.01). This indicated that the distance with the greatest wind erosion potential caused by the influence of the residence on the soil factors was 700 m. Similar changes occurred in the *COG*, reflecting that the most severe impact of human settlement on crop coverage around the residence was 500 m, and the *COG* was reduced significantly in the rest of the buffer zones. However, at a distance of 3000 m, the wind erosion potential due to crop coverage increased significantly, indicating that it might have been disturbed by other elements outside the residence. The *K’* value decreased gradually from the residences in the buffer zones of 0–600 m. However, there was a contrasting pattern, especially between 1500–3000 m, where the *K’* value showed an increasing trend. Therefore, the change characteristics of wind erosion factors were affected differently by residences.

Figure 5 also shows the trend of wind erosion factors along the road. The value of *WF* was the largest at 600 m, and the change in *WF* in the buffer zone was chaotic, which meant that the road had almost no effect on *WF*. The two factors related to soil properties, *EF* and *SCF*, both decreased significantly in the buffer zones. The difference was that *EF* rose dramatically at 600 m, while *SCF* remained at a low level. The vegetation factor *COG* follows a similar trend to the soil factors in the road buffer zones, decreasing with increasing distance. *K’* showed a climbing trend in the road buffer zones of 0–210 m. The variation beyond this range was irregular, and *K’* reached a maximum value at 600 m. This indicated that changes in soil and vegetation factors along the road buffer zone were more similar to changes in wind erosion.

### 3.3. Wind Erosion of Different Residences and Road Classes

**Figure 6:**
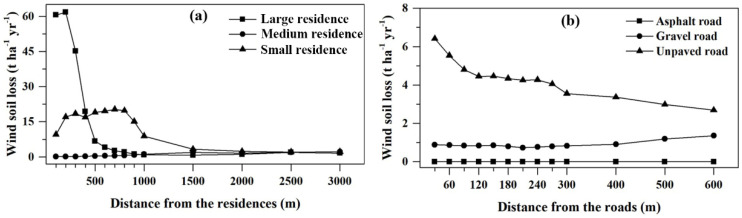
Variation in wind soil loss in the buffer zones for different sizes of residences (**a**) and types of roads (**b**). Note: large, medium, and small residences are divided according to size, corresponding to more than 4000 m^2^, 1000–4000 m^2^, and less than 1000 m^2^ respectively.

Figure 6a shows the characteristics of wind erosion variation around three classes of residences with the largest size, medium size, and smallest size distributed across the study area with varying natural environments. The differences in wind erosion among the three classes of residence sizes were reflected in the buffer zone within 1500 m, and beyond this range the wind soil loss of the three classes was very close, remaining at 0–2 t ha^−1^ yr^−1^. The large residence suffered the most severe wind erosion in the 0–200 m buffer zones, with wind erosion reaching approximately 60 t ha^−1^ yr^−1^. The wind soil loss dropped sharply to 6.76 t ha^−1^ yr^−1^ at a distance of 500 m and decreased at a slower pace than the rest of the distance to nearly 1 t ha^−1^ yr^−1^ at 600 m. For small residence, the wind soil loss was relatively strong in the 0–1000 m buffer zone and exceeded large residence in the 500 m buffer zone. However, there was a slight tendency of wind erosion variation in medium residence, and the figure remained at 0–2 t ha^−1^ yr^−1^ along with residence sizes. Therefore, the wind soil loss was related to the residence size, but this relationship was also affected by the environment in which the residences were located.

The distribution of wind erosion in the road buffer zone was closely related to the road level (Figure 6b). There was only one asphalt road in the northeastern part of the study area. The wind soil loss in the 0–600 m buffer zone of the asphalt road was close to 0. The wind soil loss in the 0–300 m buffer zone of the gravel roads remained unchanged, approximately 1 t ha^−1^ yr^−1^, and the soil loss increased slightly in the 300–600 m buffer zone. The unpaved roads with the highest network density were distributed throughout the study area. It is obvious that the buffer zones on both sides of these unpaved roads experienced strong wind erosion, especially in the range of 0–120 m; with the increase in the buffer distance of the unpaved roads, wind erosion weakened dramatically, and wind soil loss remained at 4–4.5 t ha^−1^ yr^−1^ in the buffer zone range of 120–270 m and steadily decreased to 2.7 t ha^−1^ yr^−1^ in the range of 300–600 m from the road. Therefore, compared with asphalted roads and gravel roads, the erosion around unpaved roads was much more severe, indicating that the characteristics of wind erosion were affected by road level.

## 4. Discussion

### 4.1. The Influence of Residences on Wind Erosion

Human activities around residences have caused the degradation of soil and vegetation, which has a significant impact on soil wind erosion. The characteristics of soil and vegetation in the residence buffer zone vary depending on the priority pathways of humans and livestock [30,38], and areas in the preferred pathways of humans and livestock are more vulnerable to wind erosion. The *SCF* and EF in the buffer zones 700 m away from the residence show significantly high values (Figure 5). Frequent livestock activities that feed on pastures in adjacent residence areas (Figure 1b) result in high *COG* values in the 500 m buffer zones (Figure 5). Human activities in this area lead to changes in the structure and properties of the soil. For instance, the hoof pressures exerted on the soil by animal walking are approximately 200 kPa [39], which causes a decrease in porosity [40] and an increase in bulk density [41]. Animal trampling reduces the input of above- and belowground organic matter, resulting in low organic matter and clay contents [42,43], so the *SCF* presents high values. In addition, the mechanical stress of herd trampling destroys soil aggregates [38,44], which are primarily responsible for the high *EF* values. Vegetation protects the topsoil by sheltering bare soil and extracting wind momentum [21], while the height and density of vegetation are destroyed by grazing. Therefore, human activities increase the risk of wind erosion mainly by influencing the three factors *SCF*, *EF,* and *COG*, due to the destruction of the physical and chemical properties of soil and vegetation on human priority pathways.

Generally, wind erosion is influenced by a combination of human activity and the environment. The environment, prone to erosion, is disturbed by human activities, and wind erosion is significantly increased. For example, residences with an area of more than 4000 m^2^ (Figure 6a) are adjacent to wind erosion hotspots in the central region, which has complex topography, and the buffer zones of these residences have suffered severe wind erosion under the dual effects of human activities and topography [30,32]. In comparison, an environment lacking erosive conditions can weaken the aggravation of wind erosion caused by human activities. For example, for residences of 1000–4000 m^2^ distributed in the flat west (Figure 6a), although the surrounding environments are also disturbed by human and livestock activities, the wind erosion in the buffer zones shows a slight pattern in an environment with insufficient erosive wind.

There is a superimposed effect of different residences on wind erosion. The buffer size setting is critical to the exploration of the wind erosion variability characteristics around the study object. The distance between residences is limited, and the effects of residences in the buffer zone on wind erosion interfere with each other. The center of the buffer zone of one residence may become the periphery of the buffer zone of another residence. For example, in H5 and H6, which are located in relatively densely populated western residences, as the buffer distance increases, the wind soil loss presents climbing trends with fluctuations (Figure 4a). The wind soil loss in the centers of the buffer zone of H5 and H6 is low, and the buffer zones increase outward to reach the wind erosion hotspots in the centers of H3 and H4, which causes the wind erosion in the periphery of the buffer zones of H5 and H6 to be more severe than that in the center. Large buffer distance settings result in the spatial superposition of buffers from multiple residences, while small buffer settings are not sufficient to explore spatial differences in wind erosion [33]. Although H5 and H6 in this study are slightly spatially superimposed on the other buffers, the wind erosion variability characteristics of the general trend (Figure 2) and other subjects (Figure 4a) indicate that the buffers set in this study are reasonable.

### 4.2. The Influence of Roads on Wind Erosion

The impact of the roads includes causing on-site wind erosion and increasing the sensitivity of the surrounding environment to wind erosion. The surface of roads is lower than that covered by pasture (Figure 1c). The soil of the road is exposed to strong winds without vegetation protection, and soil particles are crushed by vehicles and livestock, which accelerates the process of wind erosion on roads [23,25]. In addition, livestock affects the surrounding area of the road through trampling (Figure 1c), and both soil and vegetation factors decrease regularly along the road [33]. The impact of roads on *SCF*, *EF*, and *COG* is most significant in the 60 m buffer zone (Figure 5). This means that in the buffer zones close to the road, the soil and vegetation are destroyed due to trampling and foraging by livestock [45]. Human activities change the road and the surrounding soil and vegetation to aggravate wind erosion. Existing plot-scale studies have found that roads suffer from strong wind erosion and cause dust emissions [19,20,23]. However, there is still significant wind soil loss in the buffer zones of at least 60 m on both sides of the roads, as identified by this study (Figure 2), which has not been found by previous plot-scale studies. Therefore, considering the impact of roads on environmental soil degradation from a spatial perspective should not be ignored in ecological evaluation.

The wind erosion condition around the roads varies with the types of roads. The hardened asphalt pavement protects the road from wind erosion [24], there are fences on both sides of the road for protection, and the wind erosion in buffer zones is slight because it is rarely disturbed by livestock activities. A similar trend is seen in the gravel road, and the dust emissions of gravel roads crushed by vehicles are significantly lower than those of unpaved roads [46]. Unpaved roads lack the protection of gravel cover, and the crushed soil is exposed to strong winds. The fact that most grazing activities cross unpaved roads results in a higher density of such roads. Farming and grazing are carried out around roads, and they are frequently disturbed by humans and animals, which leads to the most serious wind erosion, and soil loss decreases with distance. In particular, unpaved roads located in wind-erosion-sensitive areas are more affected by the interference of human activities. The unpaved roads are distributed in each part of the study area, and some of the unpaved roads, as shown in Figure 1a, are distributed on windward slope areas with strong winds [47], which are prone to destructive wind erosion once they are disturbed by human activities. Therefore, wind erosion around unpaved roads in arid and semiarid environments is severe, and such unpaved roads can result in hotspots of wind erosion in landscapes.

### 4.3. The Significance of Considering Residences and Roads in Wind Erosion Evaluation

The existence of residences and roads is an important way for human activities to affect wind erosion in arid and semiarid environments. Residences and roads alter the environmental resources of the landscape and contribute to the human connection with the environment [48]. Residences are the places where humans and livestock live, and soil properties and vegetation are disturbed, such as by trampling. The intensity of wind erosion in different buffer zones is not simply related to the residence size but is affected by the dual effects of human activities and the environment, as well as the superimposed effect of the different residences. When the unpaved road is located in the wind-erosion-sensitive area, it stimulates the formation of wind erosion hotspots. The impact of the residences and roads on wind erosion is quantified from a spatial perspective based on the effective setting of the buffer size, which allows the focus of agricultural and environmental management from the point upscale to the area.

The land degradation caused by residences and unpaved roads includes two aspects: one is that residences and roads experienced severe on-site erosion [16,19]; the other is their threat of erosion to the surrounding environment, which has been ignored by many studies [26,33]. The wind erosion modelling results of this study were conducted without consideration of the residence and road indicators, which were combined with remote-sensing-image-derived residence and road distribution data to detect this objective phenomenon. The spatially overlayed analysis of these two independently acquired datasets explicitly demonstrates the crucial impacts of residences and roads on wind erosion, supporting the reliability of this study. Considering the influence of human factors such as residences and roads is of great significance to the study of the spatial variability of wind erosion because the priority pathways and intensity of human activities largely depend on the distribution of residences and roads. Human activities were used as the input data to describe the wind erosion process separately [49], and quantitatively evaluating the wind soil loss of the site and surrounding areas caused by residences and roads sheds light on spatial wind erosion modelling. Residences and roads can be included in such models as parameters that affect wind erosion in future concerns, similar to the water erosion prediction project for forest roads [50]. For example, the WEPP: Road model developed by the US Forest Service combines terrain and soil databases with GIS technology to provide a specific web-based interface for assessing forest road erosion at the watershed scale [50]. The landscape scale in wind erosion is similar to the watershed scale in water erosion, and like the WEPP: Road, erosion of residences and roads can be considered in wind erosion modelling, so that the impact of human pathways on wind erosion can be reasonably evaluated.

This study reveals that residences and roads cause severe wind erosion, and wind erosion decreases rapidly with buffer distance. Residence buffer zones are more susceptible to severe wind erosion and have a wider range of impacts than roads because residences are locations where humans and livestock have high-frequency activities. Unpaved roads are responsible for dust emissions [24] and are closely connected with residences. Hardening the cover with gravel for unpaved roads with the most frequent human activities is an effective method to effectively control wind erosion and land degradation. Moreover, the construction of residences and roads should avoid windward slopes or high-altitude wind-erosion-prone areas to avoid further aggravating wind erosion hot spots. Cautious management in arid and semiarid areas should not be limited to roads or residential sites but should also protect surrounding areas, and the findings of this study from a spatial perspective are helpful for implementing rational and effective environmental management measures for the sustainability of wind-eroded ecosystems.

## 5. Conclusions

This study is based on independent wind erosion results and remote-sensing-image-derived data to determine the impact of residences and roads on wind erosion from a spatial perspective. The main findings are as follows.

Wind erosion intensity decreases with the distances from residences and roads due to the priority pathways of human and animal activities. The wind erosion in the 0–200 m buffer zones of residences was the most serious, with a wind soil loss of 25 t ha^−1^ yr^−1^; the road experienced strong wind erosion in the 0–60 m buffer zones, with a wind soil loss of approximately 5.25 t ha^−1^ yr^−1^.

The wind erosion rate in the buffer zone was affected by the types of residences and roads, especially around unpaved roads, which were susceptible to wind erosion. Human activities around residences and roads mainly affected three wind erosion factors, *SCF*, *EF,* and *COG*, and increased wind erosion risks by destroying the physical and chemical properties of soil and vegetation on human priority pathways.

Ecological management in arid and semiarid areas should not only focus on residences and roads, but also protect surrounding environments. In future studies, residences and roads can be included in wind erosion modelling as parameters to quantitatively evaluate the wind soil loss of the site and surrounding areas.

## Figures and Tables

**Table 1:** Percentage of wind erosion intensities at different residence buffer distances.

Distances (m)	Percentage of Different Wind Erosion Intensities (%)					
	< 2 t ha^−1^ yr^−1^ (Tolerable)	2–25 t ha^−1^ yr^−1^ (Slight)	25–50 t ha^−1^ yr^−1^ (Moderate)	50–80 t ha^−1^ yr^−1^ (Severe)	80–150 t ha^−1^ yr^−1^ (Very severe)	> 150 t ha^−1^ yr^−1^ (Destructive)
30	79.36	17.37	1.06	0.29	0.38	1.55
60	79.53	17.40	0.86	0.38	0.47	1.36
90	78.28	19.04	0.72	0.41	0.51	1.05
120	77.90	19.68	0.76	0.45	0.45	0.75
150	76.66	20.54	1.07	0.51	0.50	0.71
180	75.79	21.09	1.19	0.49	0.73	0.71
210	75.45	21.92	0.95	0.32	0.80	0.57
240	75.22	22.11	0.88	0.33	0.84	0.62
270	73.88	23.49	1.01	0.18	1.09	0.35
300	72.84	24.55	1.18	0.22	0.87	0.34
400	70.33	26.90	1.44	0.53	0.80	0.00
500	62.59	35.50	1.15	0.77	0.00	0.00
600	49.85	49.18	0.97	0.00	0.00	0.00

**Table 2:** Percentage of wind erosion intensities at different road buffer distances.

Distances (m)	Percentage of Different Wind Erosion Intensities (%)					
	<2 t ha^−1^ yr^−1^ (Tolerable)	2–25 t ha^−1^ yr^−1^ (Slight)	25–50 t ha^−1^ yr^−1^ (Moderate)	50–80 t ha^−1^ yr^−1^ (Severe)	80–150 t ha^−1^ yr^−1^ (Very severe)	>150 t ha^−1^ yr^−1^ (Destructive)
30	79.36	17.37	1.06	0.29	0.38	1.55
60	79.53	17.40	0.86	0.38	0.47	1.36
90	78.28	19.04	0.72	0.41	0.51	1.05
120	77.90	19.68	0.76	0.45	0.45	0.75
150	76.66	20.54	1.07	0.51	0.50	0.71
180	75.79	21.09	1.19	0.49	0.73	0.71
210	75.45	21.92	0.95	0.32	0.80	0.57
240	75.22	22.11	0.88	0.33	0.84	0.62
270	73.88	23.49	1.01	0.18	1.09	0.35
300	72.84	24.55	1.18	0.22	0.87	0.34
400	70.33	26.90	1.44	0.53	0.80	0.00
500	62.59	35.50	1.15	0.77	0.00	0.00
600	49.85	49.18	0.97	0.00	0.00	0.00

## Data Availability

The data that support the findings of this study are available from the corresponding author upon reasonable request.
